# The Phylogeography and Spatiotemporal Spread of South-Central Skunk Rabies Virus

**DOI:** 10.1371/journal.pone.0082348

**Published:** 2013-12-03

**Authors:** Natalia A. Kuzmina, Philippe Lemey, Ivan V. Kuzmin, Bonny C. Mayes, James A. Ellison, Lillian A. Orciari, Dillon Hightower, Steven T. Taylor, Charles E. Rupprecht

**Affiliations:** 1 Rabies Program, Division of High Consequence Pathogens and Pathology, Centers for Disease Control and Prevention, Atlanta, Georgia, United States of America; 2 Department of Microbiology and Immunology, KU Leuven, Leuven, Belgium; 3 Zoonosis Control Branch, Texas Department of State Health Services, Austin, Texas, United States of America; 4 Department of Epidemiology and Public Health, Ross University School of Veterinary Medicine, Basseterre, St. Kitts, West Indies; INIAV, I.P.- National Institute of Agriculture and Veterinary Research, Portugal

## Abstract

The south-central skunk rabies virus (SCSK) is the most broadly distributed terrestrial viral lineage in North America. Skunk rabies has not been efficiently targeted by oral vaccination campaigns and represents a natural system of pathogen invasion, yielding insights to rabies emergence. In the present study we reconstructed spatiotemporal spread of SCSK in the whole territory of its circulation using a combination of Bayesian methods. The analysis based on 241 glycoprotein gene sequences demonstrated that SCSK is much more divergent phylogenetically than was appreciated previously. According to our analyses the SCSK originated in the territory of Texas ~170 years ago, and spread geographically during the following decades. The wavefront velocity in the northward direction was significantly greater than in the eastward and westward directions. Rivers (except the Mississippi River and Rio Grande River) did not constitute significant barriers for epizootic spread, in contrast to deserts and mountains. The mean dispersal rate of skunk rabies was lower than that of the raccoon and fox rabies. Viral lineages circulate in their areas with limited evidence of geographic spread during decades. However, spatiotemporal reconstruction shows that after a long period of stability the dispersal rate and wavefront velocity of SCSK are increasing. Our results indicate that there is a need to develop control measures for SCSK, and suggest how such measure can be implemented most efficiently. Our approach can be extrapolated to other rabies reservoirs and used as a tool for investigation of epizootic patterns and planning interventions towards disease elimination.

## Introduction

Rabies is a fatal zoonotic disease caused by negative-sense single-stranded RNA viruses from the genus *Lyssavirus*. Rabies virus (RABV) is the type species of the genus. It is most broadly distributed, and is the only lyssavirus documented in the New World [1]. Although canine-mediated rabies has been eradicated in the USA and Canada, wildlife rabies is still present in bats and carnivores across vast territories of the continent posing veterinary and public health risks [2].

Among carnivores affected by rabies, skunks represent a special case because of the variety of RABV variants associated with these animals [3]. Ten species of skunks are present in North America, including four species of hog-nosed skunks (genus *Conepatus* ), four species of spotted skunks (genus *Spilogale*), the hooded skunk (*Mephitis macroura*), and the most broadly distributed striped skunk (*Mephitis mephitis*) [4]. The striped skunk is the species most frequently reported rabid although the disease is also documented in the spotted, hooded and hog-nosed skunks. In many cases, diagnostic laboratories do not perform species identification, and these animals are simply registered as “skunks” [5-7].

Skunk rabies has been known in California since the beginning of the 19th century [8]. Hovey [9] proposed the name “Rabies Mephitica” for skunk disease present on the plains of Kansas and Colorado. Human rabies cases after exposures to skunks were documented in Arizona since 1875 [10]. Rabies cases in skunks decreased significantly during the first decades of the 20th century, but increased again since the late 1940s [11]. At present, skunk rabies is geographically most widespread in North America compared to the disease in other carnivores. Skunks constitute more than 30% of all “terrestrial” rabid animals documented in the USA every year [2].

Although we do not know which viruses circulated in skunks historically, the advent of monoclonal antibody and genetic typing methods demonstrated that viruses recovered from skunks since the late 1960s represent several different RABV variants [12]. These include viruses of canine origin delivered with dogs from Old World during European colonization (the north-central skunk, California skunk, Arctic RABV variant, and a viral variant encountered in skunks in Mexico), and “indigenous” American RABV lineages (another Mexican skunk RABV, and south-central skunk RABV) which are more closely related to raccoon and bat viruses [3]. In addition, several outbreaks caused by bat RABVs were documented in skunks in North America [13].

Among the skunk RABV, the south-central skunk virus variant (SCSK) is the most broadly distributed, ranging from Texas in the south to South Dakota in the north, and from Arizona in the west to Illinois in the east [2]. As was suggested, SCSK may exhibit characteristics of an epizootic virus in the Great Plains of North America based on comparative estimates of infectivity and transmissibility obtained from coupling molecular data with landscape features [14]. At least three geographically restricted phylogenetic groups of SCSK were delineated in another study [3]. Given these characteristics and lack of essential information on SCSK diversity over the broad territory of its circulation, we focused our study on this RABV lineage.

Biek et al. [15] offered a high-resolution phylogeographic model of raccoon rabies epizootic which demonstrated a good concordance with field data. This approach was recently improved to allow more robust estimations through the implementation of relaxed random walk (RRW) models [16,17] which allow reconstruction of dispersal patterns in continuous space while simultaneously reconstructing the evolutionary history from molecular sequences. We utilized this approach to reconstruct spatiotemporal patterns of SCSK based on glycoprotein (G) gene sequences of viruses sampled from the entire circulation range during more than 30 years. The G gene is one of the most variable RABV genes [18]. Even more importantly, the G is not as hyper-variable as non-coding intergenic regions of RABV, and not as constrained by purifying selection as the nucleoprotein (N) gene, the regions most frequently used in phylogenetic and molecular epidemiological studies of rabies [19,20]. Both these extremes were shown to adversely affect evolutionary estimates for various viruses [21,22]. The G gene is coding for the only outer protein of RABV, responsible for interactions with host cell receptors and recognized by host immune system [23]. Therefore, we expected that the G gene may be subjected to diversifying selection towards host adaptation and evasion of the immune system surveillance, and may be a useful marker for evolutionary analysis.

## Materials and Methods

### Samples

Brain tissue samples from rabid animals were obtained via routine surveillance activity of the Centers for Disease Control and Prevention (CDC; Atlanta, GA, USA). Institutional Animal Care and Use Committee (IACUC) or ethics committee approval was not necessary, because moribund and dead animals, or animals involved in human rabies exposure were collected by the US State Health Departments, US Department of Agriculture and veterinary laboratories during routine surveillance and diagnostic service. Authors did not implement any animal sampling during this study. The diagnosis was confirmed via the direct fluorescent antibody (DFA) test (http://www.cdc.gov/rabies/pdf/rabiesdfaspv2.pdf). Initial typing was performed via application of antinucleocapsid monoclonal antibodies [12]. The samples identified as SCSK were subsequently subjected to genetic analysis. Historical SCSK samples were retrieved from CDC archives.

Total RNA was extracted from infected brain tissues using TRIZol reagent (Invitrogen). Primers for RT-PCR were designed according to the gene sequences of rabies viruses available in GenBank. The RT-PCR was performed as described elsewhere [24]. When multiple product bands were obtained using certain primer sets, their separation in low-melting agarose gel was implemented [25]. The RT-PCR products were purified and subjected to direct sequencing. The sequencing products were processed on an ABI 3730 DNA Sequencer (Applied Biosystems). The complete G gene sequences were assembled using BioEdit 7.0.5 [26]. The dataset was supplemented with complete and partial SCSK G gene sequences available from GenBank, where isolation year and location were indicated (Table S1).

### Selection Analysis

Appropriate nucleotide substitution models were evaluated in MEGA, version 5.1 [27]. The general time-reversible model incorporating both invariant sites and a gamma distribution (GTR+I+G_4_) was favored based on both the Akaike criterion and the Bayesian information criterion. The selection analyses were performed using four methods available in the HyPhy software package (http://datamonkey.org). The single likelihood ancestor counting (SLAC) is the simplest method for assessment of selection forces. Four quantities are computed for the sequence set: normalized expected (ES and EN) and observed numbers (NS and NN) of synonymous (dS) and non-synonymous (dN) substitutions. SLAC estimates dN = NN/EN and dS = NS/ES, and if dN < (or >) dS a codon is considered negatively (or positively) selected. The fixed effect likelihood (FEL) method is a likelihood-based analogue of the site-by-site counting methods, involving estimation of dN/dS ratios for each site in a sequence alignment [28]. Both methods assume constant selection pressure over time. In contrast, the mixed effects model of episodic selection (MEME) allows for temporally varying positive selection. The MEME combines fixed effects at the level of a site with random effects at the level of branches. This model is an extension of the FEL method, where the dN/dS values are allowed to vary along branches according to a binary distribution, i.e. some branches may be under positive selection while others are under negative selection [29]. The more recently developed fast unbiased Bayes approximation (FUBAR) method utilizes a hierarchical Bayes approach that allows a flexible prior specification with no parametric constraints on the prior shape [30]. The results were considered significant at the 95% level (*p*<0.05).

### Phylogenetic analysis

Bayesian inference of time-measured evolutionary histories was performed using BEAST, version 1.7.5 [31]. In the preliminary runs we compared (using Bayes factors based on harmonic mean estimates [HME] of the marginal likelihood available in BEAST) the results obtained from a strict molecular clock versus relaxed uncorrelated log-normal molecular clock models, and the latter was favored significantly. We also found improved model fit for a partitioned model where substitution rates for the first and second codon positions (CP_1+2_) were linked, and allowed independent rates in CP_3_, versus the model where codon partitioning was set to off. We compared the exponential population growth, constant population size, Bayesian skyline, Bayesian skyride models, as well as the recently developed Bayesian skygrid model [32]. The skygrid model generalizes the Gaussian Markov random field model for population dynamic inference and provides a flexible demographic tree prior [33]. Bayesian skygrid has been shown to recover true population trajectories and TMRCA more accurately compared to other flexible coalescent models, such as Bayesian skyline and skyride [32]. All tested models produced similar Bayes factors and nearly identical evolutionary rates and divergence times for viral lineages. We selected the partitioned Bayesian skygrid model as well as the well-studied exponential population growth model for further estimates of TMRCA and substitution rates under the relaxed uncorrelated log-normal molecular clock. Two independent Markov chain Monte Carlo (MCMC) estimations were run for 70 million generations each, with samples from the posterior drawn every 7,000 generations following a burn-in of 20%. The results from the two runs were combined to generate a maximum clade credibility (MCC) tree and divergence time summaries visualized in Tracer 1.5 and FigTree 1.4 [31].

### Spatiotemporal reconstruction

To reconstruct spatial dynamics, we considered phylogenetic diffusion models in continuous space and compared strict Brownian diffusion (BD) to different RRW parameterizations [16]. To associate longitude and latitude coordinates we considered the centroids of the counties from which the samples were obtained. We compared different location diffusion models using marginal likelihood estimates obtained by importance sampling [34]. According to the HME of marginal likelihoods, the Cauchy RRW model provided the best fit. We also confirmed the diffusion model selection using more accurate stepping-stone (SS) sampling in BEAST [35].

We extracted dispersal rate and wavefront distance/velocity statistics through time from the posterior distribution by sampling each rooted phylogeny at multiple time points and summarizing the resulting distributions [17]. In brief, we sliced each rooted tree in the posterior sample at a number of points within a time interval and imputed the unobserved ancestral locations for each branch that intersects those time points [36]. We obtained the dispersal rate for each tree at each slice time by dividing the total realized dispersal path lengths, corrected for the Earth’s curvature using great circle distances (in km), since the previous time point (or since the root for the oldest slice time) by the total time elapsed along those branch paths. The wavefront distance tracks the maximum great-circle distance from the root through time for each tree, while the velocity is obtained by dividing this distance by the time since the date for the MRCA.

Summaries of the spatial spread through time were obtained using SPREAD [36] and visualized in Cartographica (http://www.macgis.com). In addition, spatiotemporal projections of phylogenies were described in the keyhole markup language (KML) for visualization in compatible geographical software such as Google Earth (http://earth.google.com).

## Results

In total, we generated complete and partial G sequences for 184 SCSK isolates (GenBank accession numbers KF484518-KF484570). We complemented this dataset with 57 SCSK G gene sequences available in GenBank with documented dates and locations. The total dataset consisted of 241 sequences (Table S1).

### Selection Analysis

The mean non-synonymous/synonymous substitution rate (dN/dS) across the SCSK dataset was 0.131 and varied from 0.033 to 0.204 in different viral lineages. All implemented methods indicated that the majority of codons were subjected to purifying selection or evolved neutrally. The results suggestive for positive selection are listed in Table 1. SLAC and FUBAR methods did not identify positively selected sites at the *p*<0.05 level. FEL and MEME identify codon 475 as positively selected site, but because it is situated in the endodomain region of RABV glycoprotein, it was not expected to influence the virus-host interactions directly. The same codon was indicated by the SLAC model below the statistical significance level (*p*=0.07). Inspection of 1000 other RABV G sequences randomly picked from GenBank indicated that R475D substitution is present in many viruses from bats, foxes, coyotes and dogs of worldwide origin (along with other substitutions in this codon as the endodomain is a highly variable region of the G). Within the G ectodomain, the only site suggested for episodic positive selection was the codon at position 184. It was identified by MEME analysis for two SCSK lineages (Table 1). The substitution G184S was present in viruses from these lineages quite consistently albeit not exclusively. Inspection of additional sequences from GenBank indicated that both G and S in the position 184 are present in glycoproteins of the raccoon, SCSK, and Mexican skunk RABV lineages, whereas other viruses associated with bats and carnivores (including such viruses associated with skunks as north-central and California skunk variants) usually harbor R_184_. Therefore, substitutions R184G/S do not appear to be species-specific. 

**Table 1:** Results of the positive selection analysis of SCSK.

Method of the analysis	Codon positions (signal peptide sequence deleted)	Amino acid substitutions and corresponding viral lineages in the phylogenetic tree (Figure 1)
SLAC	No positively selected sites	
FEL	475	R/G in the node of the MO lineage, and on the tips within TX3a and GP lineages.
MEME	184	G/S in the nodes of the MO and TX2a lineages.
	475	R/G in the node of the MO lineage, and on the tips within TX3a and GP lineages.
FUBAR	No positively selected cites	

### Phylogenetic analysis

**Figure 1:**
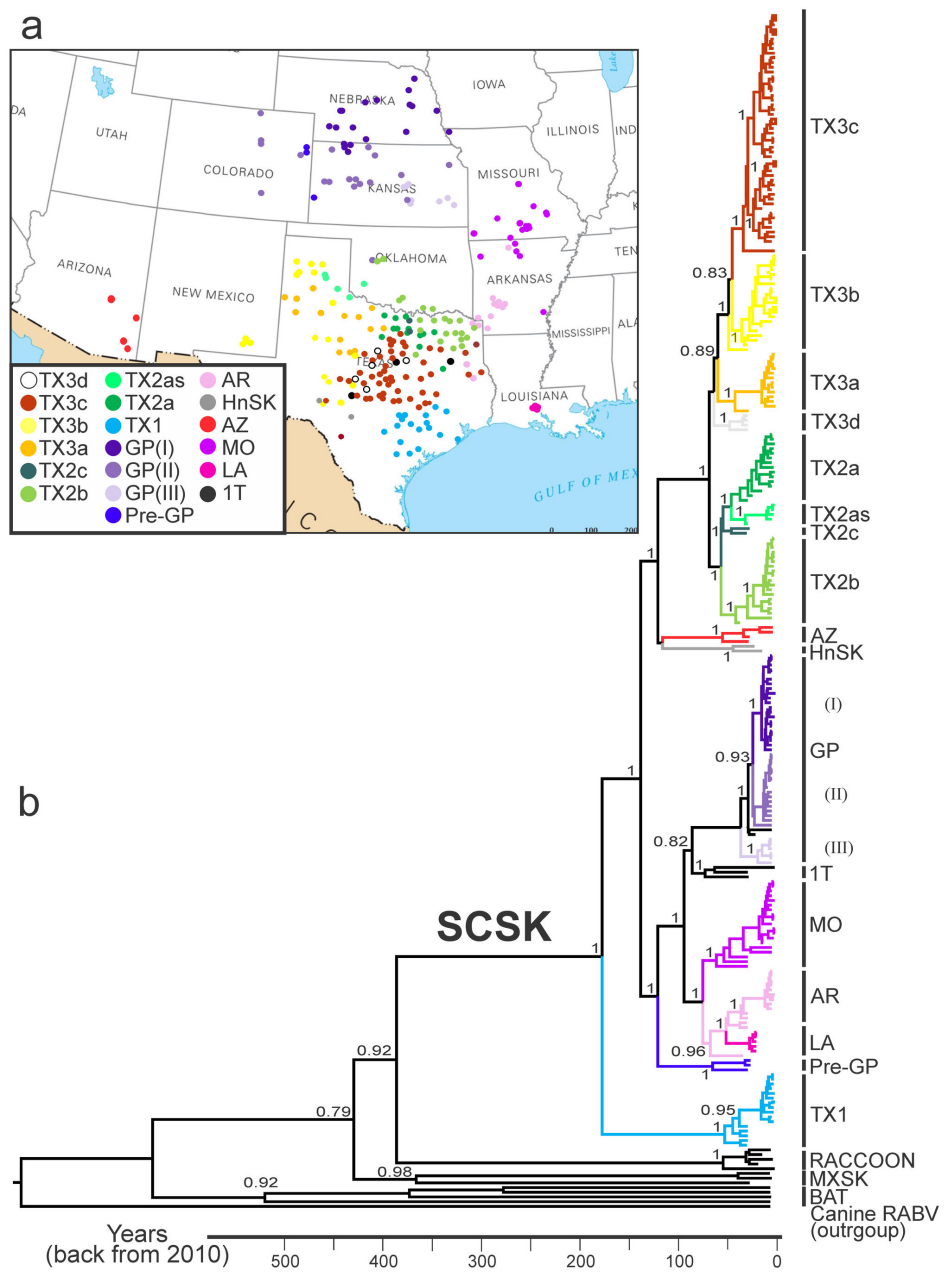
Map (a) and maximum clade credibility tree (b) of SCSK G gene sequences used in the study. Posterior probabilities are shown for key nodes, and timescale is present at the bottom. To show the phylogenetic position of SCSK in the context of other related RABV lineages, several representative G gene sequences of raccoon RABV (RACCOON), north-central/central Mexican skunk RABV (MXSK), bat RABV (BAT) are shown, and a canine RABV is used as an outgroup.

Equal tree topologies were inferred using Bayesian genealogical reconstruction under parametric and non-parametric demographic tree priors (Figure 1) and the estimates of substitution rates and time of the most recent common ancestor (TMRCA) for viral lineages were nearly identical (Table 2). The raccoon and Mexican skunk RABV lineages were the most closely related lineages to the SCSK. The raccoon RABV was separated from SCSK by a long branch, whereas Mexican skunks represented quite diverse viruses of ancient origin. Even greater distances separated all these “terrestrial” RABV lineages from bat RABV lineages.

**Table 2:** Characteristics of the SCSK phylogenetic lineages identified in the present study.

Lineage	No of sequences	Year span	dN/dS	Substitution rate*	TMRCA with 95% HPD in the brackets*
1T	3	1984-2012	0.11	3.23E-4	73 (58-91)
AR	12	1979-2009	0.09	3.32E-4	68 (59-81)
AZ	4	1985-2010	0.17	4.12E-4	55 (42-70)
HnSK	2	1991-1999	NA†	2.21E-4	44 (31-61)
GP	45	2005-2012	0.14	2.63E-4	36 (27-45)
LA	7	1983-1993	0.10	3.82E-4	50 (42-59)
MO	19	1984-2012	0.10	3.65E-4	62 (50-74)
Pre-GP	3	1984-1987	0.09	3.05E-4	65 (49-85)
TX1	16	1983-2011	0.08	3.23E-4	53 (43-65)
TX2a	15	1984-2012	0.08	3.71E-4	37 (31-43)
TX2as	5	1984-2012	0.08	3.11E-4	31 (29-37)
TX2b	19	1976-2012	0.03	3.53E-4	41 (37-46)
TX2c	2	1983-1986	NA†	3.11E-4	46 (37-55)
TX3a	13	1985-2013	0.15	3.39E-4	42 (33-51)
TX3b	20	1984-2012	0.07	2.95E-4	35 (31-40)
TX3c	51	1985-2012	0.06	3.72E-4	34 (28-39)
TX3d	5	1984	0.09	3.11E-4	48 (38-58)
All SCSK lineages	241	1979-2013	0.13	4.12E-4	171 (132-212)

* Calculated from the whole SCSK tree.

† Not available due to a limited sequence number.

The SCSK viruses were segregated in 17 lineages circulating in specific geographic territories. Viruses from Texas (TX1, TX2a-c, TX3a-d, and 1T) were most divergent, representing both basal and more recently diverged lineages. The lineage TX1, is basal for all other SCSK lineages that were further subdivided in two large groups. One group of viruses gave the origin for lineages circulating northward and eastward from Texas (Pre-GP, LA, AR, MO, and GP), whereas the other group continued to evolve locally in Texas (lineages TX2a-c, TX3a-d, and HnSK) and spread westward to Arizona (lineage AZ).

As a general rule, each lineage contained viruses isolated within a 20-30 year span, with older viruses occupying basal positions within each lineage. The exceptions from this general rule included the lineage TX3d which contained only old viruses (isolated in 1984) and was an ancestor for lineages TX3a-c (although each of the latter contained both old and recent viruses, indicating that diversification from the TX3d lineage had occurred earlier). Another exception was the lineage Pre-GP that contained viruses from Colorado and Kansas obtained during 1984-1987, and was an basal for lineages LA, MO, 1T and GP. The lineage GP was also subdivided into 3 territorial clusters, I-III, but genetic distances between these were very short, reflecting <2.1% nucleotide divergence, whereas nucleotide divergence between the major SCSK lineages (supported with geographical diversification) ranged 3.6-4.9%.

The TMRCA of SCSK viruses was estimated at 171 years (95% HPD 132-212). Substitution rates ranged from 2.21 x 10^-4^ to 4.12 x 10^-4^ which corresponded to estimates provided for various RABV G sequences elsewhere [18,37].

### Spatiotemporal reconstruction

**Figure 2:**
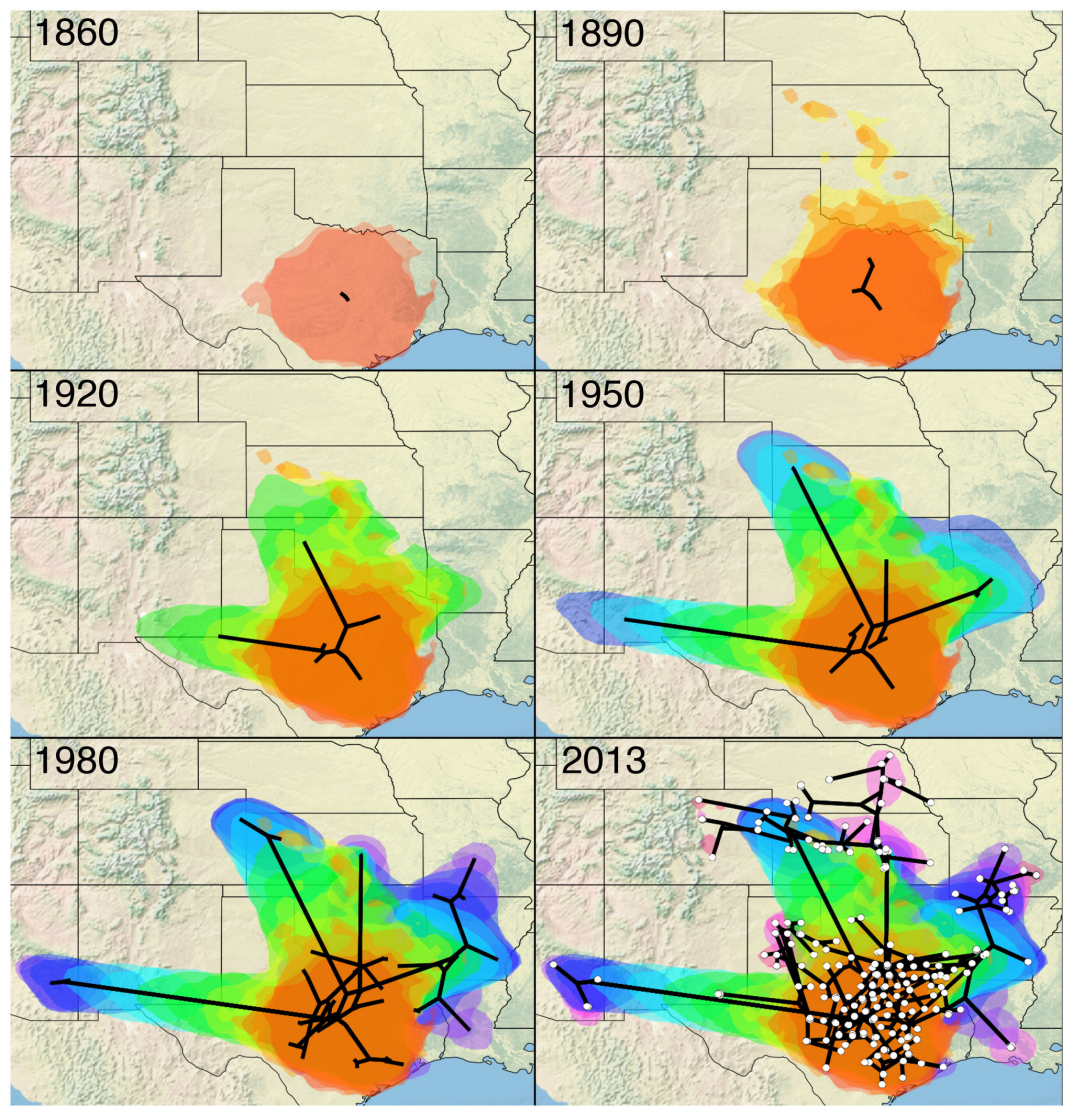
The reconstructed spatiotemporal diffusion of SCSK at different time points from 1860 to 2013. Black lines show a spatial projection of a representative phylogeny, with each node being mapped to its known (external node) or estimated (internal node) location. In each panel colored clouds (cumulative, in different colors for each state) represent statistical uncertainty in the estimated locations of the SCSK lineages (95% HPD regions). In the last panel (2013), white circles indicate isolate sampling locations.

Combining the molecular sequence data with isolation times and geographical coordinates, a spatiotemporal distribution of SCSK was inferred (Figure 2). According to the basal position of the TX1 lineage, an ancestor of the presently circulating SCSK viruses existed in Texas during the first half of the 19th century. This origin is also supported by the greatest diversity of SCSK lineages currently present in the territory of Texas. These lineages occupy specific geographic areas with very limited overlaps although inspection of multi-layered geographic maps did not allow identification of natural or artificial barriers restricting virus spread. In addition, lineages TX2as and TX2b are present throughout central Oklahoma, whereas only one virus from lineage GP was recovered in the northern part of this state.

Moving northward, by the end of the 19th century the epizootic affected Kansas. Viruses of the same origin spread over lowland territories of Arkansas, Missouri and Louisiana during the first half of 20th century, reaching the Mississippi River which constituted a major barrier for epizootic spread. It is interesting that one group of viruses originating from the same ancestor (lineage 1T) was present in Texas at least during 1984-2012. Compared to the diversity of SCSK lineages in the southern part of the virus range, lineage GP that circulates in vast territories of the Great Plains is very homogenous even if it can be subdivided in three territorial clusters I-III.

Another lineage of SCSK expanded westward, crossing the southern part of New Mexico and reaching southern Arizona by 1950. Mountains constitute barriers for epizootic spread in New Mexico, Arizona and western Colorado. The Mojave Desert and the Sonoran Desert limit further SCSK dispersal to the west (and suppress the eastward spread of the California skunk RABV).

**Figure 3:**
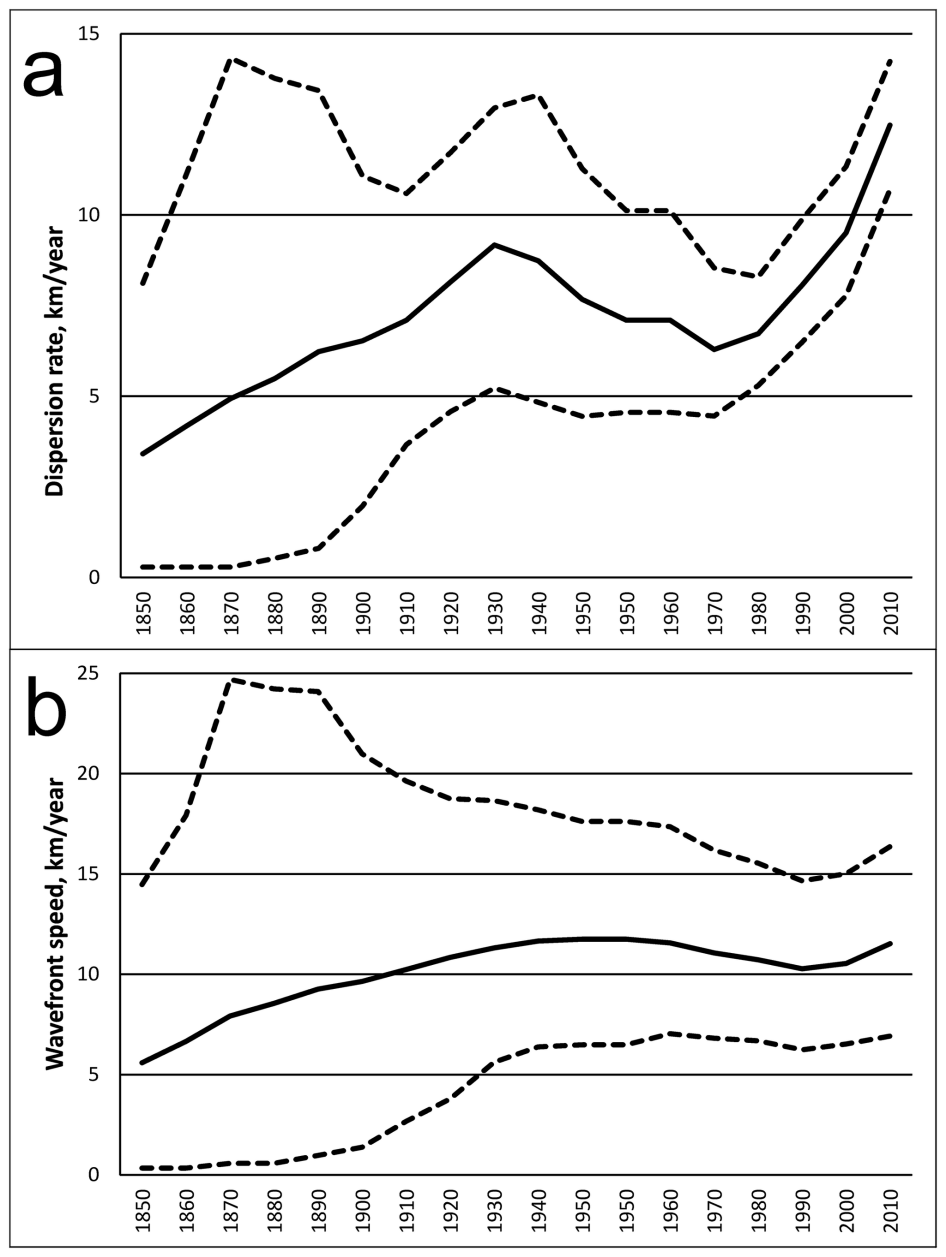
The reconstruction of spatiotemporal dynamics of SCSK inferred from the Bayesian skygrid model: dispersal rate (a) and wavefront velocity (b). Years are shown on the horizontal axis. The dashed lines indicate 95% HPD.

Mean dispersal rate of SCSK was 9.45 km/yr, and mean wavefront velocity was 10.04 km/yr. Spatiotemporal limitations of the dataset did not allow robust comparisons of these parameters for different SCSK lineages, however we were able to perform estimations for northward, westward and eastward directions of SCSK spread, as compared to the location of the tree root (Table 3). The wavefront velocity in the northward direction was significantly greater than the velocities in westward and eastward directions. The dispersal rate for the northward direction was also greater, although not statistically significant. In addition, these parameters were not constant over time (Figure 3). The first increase in the dispersal rate occurred during 1850-1930, followed by a slightly decreasing plateau during 1940-1980, and by another growth period from ~1980 to date. The wavefront velocity changed more smoothly, with a slight increase occurred during 1850-1940, followed by a plateau during 1950-1990, and another steady increase thereafter.

**Table 3:** Spatiotemporal parameters of SCSK estimated using relaxed random walk models (km/yr), mean and 95% HPD in the brackets.

Spread direction*	Dispersal rate	Wavefront velocity
North	13.14 (10.74-16.04)	10.52 (8.91-12.13)
West	10.54 (7.15-13.36)	8.04 (7.43-8.65)
East	8.66 (6.65-10.72)	8.46 (7.24-9.74)
Whole SCSK	9.45 (8.15-10.58)	10.04 (9.12-10.96)

* The directions of SCSK spread were determined compared to the location of the tree root, and after removal of lineage 1T (which probably migrated in a backward direction). For the whole SCSK, all sequences were included.

## Discussion

In the present study we reconstructed phylogeography and spatiotemporal history of one of the major “indigenous” American RABV lineages, SCSK. Available surveillance records demonstrate that our sampling encompassed all areas of contemporary SCSK circulation in the US [2]. Oral rabies vaccination (ORV) of skunks has not been consistently implemented in the US to date [38]. The lack of intervention measures means that skunk rabies can be viewed as a natural process of pathogen invasion yielding insights to disease emergence. 

We demonstrated that SCSK is genetically much more divergent than was inferred previously [3,14]. Evolutionary analysis did not show substantial evidence for positive selection in the dataset. In general, this is typical for RABV genes. In several previous studies a positive selection was documented inconsistently in RABV genes, and codons suggested for positive selection varied depending on datasets analyzed and methods used [13,18,39-41]. Furthermore, we conclude that results of MEME analysis should be interpreted cautiously as regard to the species adaptation, even if episodic adaptive selection has been detected. 

The most significant diversity of SCSK was observed in Texas, and at least one of the Texas lineages (TX1) was placed at the base of the phylogenetic tree. Such phylogenetic patterns suggest that SCSK originated in the territory of Texas, although historical introduction of an ancestral virus from Mexico still cannot be ruled out based on the available samples. Phylogenetically related viruses circulate in skunks in Mexico, the so-called central or north-central Mexican skunk RABV lineages [3,42]. However, no SCSK isolates from Mexico are available in GenBank and our archives. Moreover, surveillance records indicate that SCSK has been absent in southern Texas along the Mexican border for many years [2,43]. These observations suggest that arid landscapes of this area and the Rio Grande River represent barriers for virus spread.

Viruses from the SCSK lineages present in Texas perpetuate in their limited geographic areas. Studying raccoon rabies, Biek et al. [15] found that major diversification of viruses occurred shortly after the initial infection wave. Counties sampled 5-25 years after the first wave consistently yielded the same genetic lineage that had colonized the area initially. In our study, all SCSK lineages from Texas were localized geographically during 5-20 years of sampling, with older sequences present at the bases or inside the tree clades. This observation implies that colonization of the territory had occurred at least several years before sampling. Moreover, circulation areas of the lineages had only limited overlaps, although we could not identify any obvious natural barriers restricting virus spread (Figure 1). Local perpetuation of RABV is also supported by surveillance records which describe outbreaks of skunk rabies as limited foci with increased disease incidence but irregular local geographic spread [6,43,44]. Ecological studies demonstrated that skunks reach higher concentrations and live more sedentarily in proximity to humans, where they can find a variety of food. Skunk home range is usually no larger than 0.5-1.6 km^2^ in urban areas, but may extend to 3-5 km^2^ in rural areas [45]. In addition, these animals have demonstrated a limited mobility travelling ~2 km per night [5,45,46]. Monitoring of radio-collared skunks during a rabies epizootic suggested that rabid animals did not exhibit increased mobility as compared to healthy individuals [46]. 

Biek et al. [15] estimated dispersal speed for raccoon rabies as 38.4 km/yr during the initial epizootic wave and 9.5 km/yr at later stages. Even greater rates, 35-50 km/yr, were documented for fox rabies in Europe prior to implementation of ORV programs [47,48]. Our estimates suggested that the velocity of the SCSK dispersal varied between 4-12 km/yr (Figure 3). Such comparatively low values are likely associated with ecological characteristics of the host (limited mobility and contact rates) and virus-host interaction traits (pathogenicity, occurrence of the virus in salivary glands, and transmissibility) which need to be addressed in further studies. As for barriers, highways and rivers do not suppress gene flow in skunk populations [49]. We did not observe association of these landscape features with geographical compartmentalization of SCSK lineages, including the Red River and the Arkansas River. The only exceptions were the Mississippi River which limits the eastward spread of the virus and the Rio Grande River which could limit the southward virus spread to date. 

The SCSK lineages from northern and western territories are younger and less divergent than in Texas. In fact, the GP lineage from vast territories of northern Oklahoma, Kansas, western Colorado and Nebraska is genetically very homogenous. We hypothesize that skunks in rural areas of Great Plains have larger home ranges and greater mobility, so that an epizootic wave could cover broad area during a relatively short time, with a limited number of virus passages. This is supported with the greater wavefront velocity in the northward direction (Table 3) and is concordant with the “surfing mutation” model [50,51]. Based on the phylogeny, a possibility exists that viruses from lineage 1T were delivered from Great Plains back to Texas. Such reverse spread might occur via a secondary epizootic wave or via a long-distance translocation (although we did not find suggestions for such event in available literature). Conversely, this lineage might evolve in Texas. Geographic representation of viral clusters I-III within lineage GP (Figure 1) supports the hypothesis that SCSK was moving to Great Plains from the south. In this context we can speculate that ancestral viruses from lineage Pre-GP, available in our study from western Kansas and Colorado only, historically circulated more broadly, including the southern areas. Accordingly, if Pre-GP viruses circulated also in Texas, the lineage 1T could evolve locally rather than being introduced back to Texas from Great Plains.

We reconstructed the spatiotemporal history of the SCSK epizootic using phylogenetic diffusion models. The robustness of such an approach was verified previously on examples of other epizootics, where sampling covered the initial infection waves and a number of years thereafter [16,17]. Our model suggests that the MRCA of the present SCSK originated ~170 years ago. We do not know which RABVs were present in skunks in Arizona, Kansas and Colorado during the 19th century [9,10]. That could be SCSK (in such case our model does not cover the initial epizootic wave) or other RABV variants. As indicated by Charlton et al. [8], skunk rabies almost disappeared in these states during the early decades of 20th century, with new cases increasingly documented since the 1940s. 

A time-homogenous process of dispersal is likely to be an unrealistic approximation of viral dispersion, and implementation of RRW models significantly improves robustness of the spatial inference [16]. According to our estimations, the dispersal rate of SCSK increased (likely corresponding to the initial infection wave) until ~1930, following by a period of stability during 40-50 years, and subsequently by a new increase since ~1980, which continues to date (Figure 3). Moreover, after a long period of stability, the wavefront velocity of SCSK increased during the last decade as well. Our sampling covers the period of the 1980s-2000s, and our model for this period of time is most precise. Our model implies that SCSK exhibits a second large-scale expansion at the present time. A similar dynamic was observed for the mid-Atlantic raccoon rabies epizootic [15]. We do not know the reasons for such large-scale changes in epizootic process, but they may be associated with global variations in host populations, associated with, for example, climate changes, human population expansion, agricultural developments, or other large-scale factors. Alternatively, they can be caused by changes in the pathogen. But we did not identify diversifying selection in SCSK G genes sampled during the last >30 years, and it is unlikely that changes in other viral genes (coding for internal proteins) might have such significant effect on virus-host interactions.

The SCSK circulation range is greater than circulation ranges of other skunk-associated RABV variants. Studying patterns of sympatric circulation of SCSK and north-central skunk (NCSK) RABV in the territory of the Great Plains, Barton et al. [14] suggested that SCSK may have an increased pathogenicity compared to the NCSK. However, such suggestions should be corroborated via appropriate biological experiments. The NCSK seems younger than SCSK, as is suggested by our preliminary estimates (Figure S1), but this hypothesis needs additional corroboration from further studies. At this time, we do not have a sufficiently representative dataset (per space and time) of NCSK gene sequences to perform a meaningful evolutionary comparison. 

Our results demonstrate that the SCSK epizootic follows the same major patterns that were implied for the well documented mid-Atlantic raccoon rabies epizootic [15,16] albeit with a lower dispersion speed, and suggest that SCSK will extend its range during the near term. The most significant spread is expected in the northern direction. Eastward expansion is also possible, once the virus crosses the Mississippi River. As the dispersal of SCSK is relatively slow, and viral lineages have occupied the same territories for decades without significant geographical spread, such territories could be targeted by local or step-wise ORV programs. One strategic area for initiation of such a program might be the Mississippi River valley. 

We believe that our approach and findings can be extrapolated to other rabies reservoirs, particularly considering a potential risk of re-introduction of canine-mediated rabies in the US [38], host shifts of RABV [13], or invasion of canine rabies in wildlife populations elsewhere [52], and used as a tool for investigation of epizootic patterns and planning interventions towards disease elimination.

## Supporting Information

Table S1 **SCSK gene sequences used in the present study.**
(DOCX)

Figure S1 **Preliminary estimates of the TMRCA for several North American RABV lineages based on complete and partial G gene sequences.** The SCSK, raccoon (RAC), and Mexican skunk (MexSK) clusters include the same sequences that were used in this study (Figure 1). Bat viruses (BAT) include several representative viral sequences from various bat species. The north-central skunk (NCSK) cluster includes 35 dated viral sequences available from GenBank or generated de novo.
(TIF)
