# Supplementary material for: The Phylogeography and Spatiotemporal Spread of South-Central Skunk Rabies Virus
Source: PLoS One. 2013 Dec 3;8(12):e82348. doi: 10.1371/journal.pone.0082348 (PMC3849458; doi:10.1371/journal.pone.0082348)
Supplement: Table S1 — SCSK gene sequences used in the present study. (DOCX) [file pone.0082348.s001.docx]

Table S1. SCSK gene sequences used in the present study

| **Sequence name** | **Phylogenetic lineage** | **Isolation year** | **County or ZIP code** | **State** | **Animal species** | **GenBank accession No** |
| --- | --- | --- | --- | --- | --- | --- |
| A10-0514 | AZ | 2010 | Cochise | AZ | Skunk* | JQ685938 |
| A10-0512 | AZ | 2010 | Cochise | AZ | Skunk | JQ685968 |
| A11-3020 | TX1 | 2011 | Jackson | TX | Skunk | JQ685984 |
| A09-0255 | TX2b | 2009 | McCurtain | OK | Domestic dog | JQ685991 |
| A08-0500 | GP | 2008 | Denver | CO | Domestic cat | JQ685993 |
| A08-0501 | GP | 2008 | Denver | CO | Raccoon | KC791812 |
| A09-5803 | TX1 | 2009 | Goliad | TX | Skunk | KC791826 |
| A09-5805 | TX2a | 2009 | Denton | TX | Skunk | KC791827 |
| A09-5807 | TX2b | 2009 | Fannin | TX | Skunk | KC791828 |
| A09-5809 | TX2a | 2009 | Dallas | TX | Skunk | KC791829 |
| A09-5811 | TX3c | 2009 | Gregg | TX | Skunk | KC791830 |
| A09-5813 | TX1 | 2009 | Colorado | TX | Skunk | KC791831 |
| A09-5815 | TX3c | 2009 | Coryell | TX | Skunk | KC791832 |
| A09-5817 | TX3c | 2009 | Coryell | TX | Skunk | KC791833 |
| A09-5819 | AR | 2009 | Bowie | TX | Skunk | KC791834 |
| A09-5821 | TX3c | 2009 | Brazos | TX | Skunk | KC791835 |
| A09-5823 | TX3c | 2009 | Brazos | TX | Skunk | KC791836 |
| A09-6175 | TX3c | 2009 | Limestone | TX | Skunk | KC791837 |
| A09-6177 | TX3b | 2009 | Scurry | TX | Skunk | KC791838 |
| A09-6178 | TX3c | 2009 | Brazos | TX | Skunk | KC791839 |
| A09-6179 | TX2a | 2009 | Denton | TX | Skunk | KC791840 |
| A09-6180 | TX3b | 2009 | Randall | TX | Skunk | KC791841 |
| A09-6181 | TX2b | 2009 | Collin | TX | Skunk | KC791842 |
| A09-6182 | TX3c | 2009 | Wise | TX | Skunk | KC791843 |
| A09-6183 | TX3b | 2009 | Tom Green | TX | Skunk | KC791844 |
| A09-6184 | TX3c | 2009 | Tarrant | TX | Skunk | KC791845 |
| A09-6185 | TX3b | 2009 | Randall | TX | Skunk | KC791846 |
| A09-6186 | TX2a | 2009 | Hunt | TX | Skunk | KC791847 |
| A11-1641 | TX3b | 2011 | Gray | TX | Skunk | KC791897 |
| A11-1642 | TX3c | 2011 | Williamson | TX | Skunk | KC791898 |
| A11-1643 | TX3a | 2011 | Briscoe | TX | Skunk | KC791899 |
| A11-1644 | TX3c | 2011 | Tarrant | TX | Skunk | KC791900 |
| A11-1645 | TX3c | 2011 | Coryell | TX | Skunk | KC791901 |
| A11-1646 | TX2a | 2011 | Wise | TX | Skunk | KC791902 |
| A11-1647 | TX3a | 2011 | Callahan | TX | Skunk | KC791903 |
| A11-1648 | TX3c | 2011 | Parker | TX | Skunk | KC791904 |
| A11-1837 | MO | 2011 | Dent | MO | Skunk | KC791911 |
| A11-1838 | MO | 2011 | Howell | MO | Skunk | KC791912 |
| A11-1839 | MO | 2011 | Howell | MO | Skunk | KC791913 |
| A11-2602 | TX3c | 2011 | Parker | TX | Skunk | KC791916 |
| A11-2603 | TX3c | 2011 | Parker | TX | Skunk | KC791917 |
| A11-2605 | TX3c | 2011 | Hamilton | TX | Skunk | KC791918 |
| A11-2608 | TX3c | 2011 | Sutton | TX | Skunk | KC791919 |
| A11-2611 | TX3c | 2011 | San Sabe | TX | Skunk | KC791920 |
| A11-2613 | TX1 | 2011 | Bastrop | TX | Skunk | KC791921 |
| A11-2614 | TX3c | 2011 | McLennan | TX | Skunk | KC791922 |
| A11-2616 | TX2b | 2011 | Hopkins | TX | Skunk | KC791924 |
| A11-2617 | TX2b | 2011 | Red River | TX | Skunk | KC791925 |
| A11-2618 | TX2b | 2011 | Wood | TX | Skunk | KC791926 |
| A11-2619 | TX3c | 2011 | Hamilton | TX | Skunk | KC791927 |
| A11-2620 | TX2a | 2011 | Rockwell | TX | Skunk | KC791928 |
| A11-2621 | TX3c | 2011 | Burnet | TX | Skunk | KC791929 |
| A11-2623 | TX2as | 2011 | Childress | TX | Skunk | KC791930 |
| A11-2626 | TX3c | 2011 | Williamson | TX | Skunk | KC791931 |
| A11-2628 | TX3c | 2011 | Mason | TX | Skunk | KC791932 |
| A11-2629 | TX2a | 2011 | Denton | TX | Skunk | KC791933 |
| A11-2632 | TX3c | 2011 | Burnet | TX | Skunk | KC791935 |
| A11-2633 | TX2b | 2011 | Lamar | TX | Skunk | KC791936 |
| A11-2634 | TX3a | 2011 | Wichita | TX | Skunk | KC791937 |
| A11-2997 | TX3c | 2011 | McCulloh | TX | Skunk | KC791938 |
| A11-2630 | TX3c | 2011 | Burnet | TX | Skunk | KC791939 |
| A11-2998 | TX3c | 2011 | Erath | TX | Skunk | KC791939 |
| A11-2999 | TX2a | 2011 | Wise | TX | Skunk | KC791940 |
| A11-3001 | TX3c | 2011 | Brown | TX | Skunk | KC791941 |
| A11-3002 | TX3c | 2011 | Llano | TX | Skunk | KC791942 |
| A11-3003 | TX3c | 2011 | Bell | TX | Skunk | KC791943 |
| A11-3004 | TX3a | 2011 | Hale | TX | Skunk | KC791944 |
| A11-3005 | TX1 | 2011 | Guadalupe | TX | Skunk | KC791945 |
| A11-3008 | TX1 | 2011 | Bastrop | TX | Skunk | KC791946 |
| A11-3009 | TX3b | 2011 | Potter | TX | Skunk | KC791947 |
| A11-3010 | TX1 | 2011 | Washington | TX | Skunk | KC791948 |
| A11-3011 | TX3c | 2011 | Hood | TX | Skunk | KC791949 |
| A11-3012 | TX3a | 2011 | Callahan | TX | Skunk | KC791950 |
| A11-3016 | TX2a | 2011 | Cooke | TX | Skunk | KC791951 |
| A11-3018 | TX3c | 2011 | Hamilton | TX | Skunk | KC791952 |
| A11-3019 | TX3c | 2011 | Comanche | TX | Skunk | KC791953 |
| A11-4061 | TX3b | 2011 | Armstrong | TX | Skunk | KC791968 |
| A11-4071 | TX1 | 2011 | Austin | TX | Skunk | KC791969 |
| A11-5276 | TX3c | 2011 | Somervell | TX | Skunk | KC792008 |
| A11-5277 | TX3c | 2011 | Eastland | TX | Skunk | KC792009 |
| A11-5279 | TX1 | 2011 | Fayette | TX | Skunk | KC792010 |
| A11-5282 | TX3c | 2011 | Llano | TX | Skunk | KC792011 |
| A11-5284 | TX3a | 2011 | Dickens | TX | Skunk | KC792012 |
| A11-5285 | TX2a | 2011 | Montague | TX | Skunk | KC792013 |
| A11-5287 | TX3a | 2011 | Jones | TX | Skunk | KC792014 |
| A11-5291 | TX3b | 2011 | Carson | TX | Skunk | KC792015 |
| A11-5293 | TX3a | 2011 | Jack | TX | Skunk | KC792016 |
| A11-5702 | MO | 2011 | Howell | MO | Skunk | KC792024 |
| A11-5703 | MO | 2011 | Howell | MO | Skunk | KC792025 |
| A11-6353 | TX3c | 2011 | Burleson | TX | Skunk | KC792038 |
| A11-6362 | TX2as | 2011 | Donley | TX | Skunk | KC792039 |
| A12-0575 | MO | 2012 | Howell | MO | Skunk | KC792053 |
| A12-0576 | MO | 2012 | Reynolds | MO | Skunk | KC792054 |
| A12-0577 | MO | 2012 | Reynolds | MO | Skunk | KC792055 |
| A12-1010 | TX2a | 2012 | Collin | TX | Skunk | KC792062 |
| A12_1012 | 1T | 2012 | Smith | TX | Skunk | KC792063 |
| A12-1018 | TX2b | 2012 | Upshure | TX | Skunk | KC792064 |
| A12-1019 | TX3c | 2012 | Gillespie | TX | Skunk | KC792065 |
| A12-1021 | TX2b | 2012 | Grayson | TX | Skunk | KC792066 |
| A12-1034 | TX3b | 2012 | Carlsbad | NM | Skunk | KC792069 |
| A12-1035 | TX3b | 2012 | Carlsbad | NM | Gray fox | KC792070 |
| A12-1748 | TX3b | 2012 | Eddy | NM | Gray fox | KC792075 |
| A12-1886 | TX3c | 2012 | Mills | TX | Skunk | KC792086 |
| A12-1893 | TX3b | 2012 | Sutton | TX | Skunk | KC792087 |
| A12-1894 | TX1 | 2012 | Grimes | TX | Skunk | KC792088 |
| A12-1898 | TX2b | 2012 | Franklin | TX | Skunk | KC792089 |
| A12-1906 | TX2a | 2012 | Palo Pinto | TX | Skunk | KC792090 |
| A12-1988 | GP | 2012 | Fort Collins | CO | Skunk | KC792100 |
| A12-2102 | TX3b | 2012 | Carlsbad | NM | Domestic dog | KC792104 |
| A12-2499 | TX2as | 2012 | Donley | TX | Skunk | KC792114 |
| A12-2500 | TX3a | 2012 | Lamb | TX | Skunk | KC792115 |
| A12-2501 | TX3c | 2012 | Robertson | TX | Skunk | KC792116 |
| A12-2505 | TX2b | 2012 | Morris | TX | Skunk | KC792117 |
| A12-2506 | TX3c | 2012 | Concho | TX | Skunk | KC792118 |
| A12-2511 | TX3c | 2012 | Millam | TX | Skunk | KC792119 |
| A12-2512 | TX3b | 2012 | Cottle | TX | Skunk | KC792120 |
| A12-2513 | TX3b | 2012 | Fisher | TX | Skunk | KC792121 |
| A12-5923 | TX3c | 2012 | Nacogdoches | TX | Skunk | KC792136 |
| A12-5924 | AR | 2012 | Marion | TX | Skunk | KC792137 |
| A12-5925 | TX3c | 2012 | Kaufman | TX | Skunk | KC792138 |
| A12-5928 | TX3c | 2012 | Travis | TX | Skunk | KC792139 |
| A12-5930 | TX3a | 2012 | Taylor | TX | Skunk | KC792140 |
| A12-5935 | TX3c | 2012 | Shelby | TX | Skunk | KC792141 |
| A12-5936 | TX2b | 2012 | Titus | TX | Skunk | KC792142 |
| A12-9362 | TX3c | 2012 | Madison | TX | Skunk | KC792146 |
| AR1081 | AR | 1984 | Hempstead | AR | Skunk | KC792191 |
| AR653 | MO | 1984 | Desha | AR | Skunk | KC792193 |
| LA179 | LA | 1993 | Lafayette | LA | Skunk | KC792209 |
| LA2210 | LA | 1993 | Lafayette | LA | Skunk | KC792210 |
| LA3034 | LA | 1993 | Lafayette | LA | Skunk | KC792211 |
| LA492 | LA | 1993 | Lafayette | LA | Skunk | KC792212 |
| SK2209 | LA | 1993 | Lafayette | LA | Skunk | KC792213 |
| MO0691 | MO | 1984 | McDonald | MO | Domestic dog | KC792215 |
| MO294 | MO | 2000 | Green | MO | Domestic cat | KC792217 |
| NESK6022 | GP | 2005 | Norfolk | NE | Skunk | KC792237 |
| OK1130 | TX2b | 1976 | Bryan | OK | Unknown | KC792239 |
| TX1327 | hnsk | 1991 | Sutton | TX | Hognosed skunk | KC792266 |
| TX4137 | TX3b | 1984 | Kimble | TX | Skunk | KC792267 |
| TX4380 | TX2c | 1983 | Cooke | TX | Spotted skunk | KC792268 |
| sm4704 | TX3c | 1999 | Freestone | TX | Domestic cat | KC792269 |
| TX4546 | TX3c | 1985 | Coryell | TX | Spotted skunk | KC792269 |
| TX5046 | 1T | 1985 | Mason | TX | Skunk | KC792270 |
| TX5328 | AR | 1983 | Rusk | TX | Skunk | KC792271 |
| TX5171 | AR | 1984 | Bowie | TX | Skunk | KC792274 |
| A11-3021 | TX3c | 2011 | Navarro | TX | Skunk | KF484560 |
| OKbull19 | TX2a | 1989 | Murray | OK | Bull | KF484542 |
| sm4710 | hnsk | 1999 | Kerr | TX | Domestic cat | KF484547 |
| sm4712 | TX2a | 1999 | Cook | TX | Domestic cat | KF484548 |
| sm4708 | TX3b | 1999 | Callahan | TX | Domestic cat | KF484546 |
| sm781 | TX3a | 1985 | Knox | TX | Domestic dog | KF484558 |
| sm795 | TX3d | 1984 | Erath | TX | Domestic dog | KF484570 |
| KS1552 | KS | 1992 | Marion | KS | Domestic dog | KF484539 |
| A13-3210 | AZ | 2013 | Pima | AZ | Hooded skunk | KF484525 |
| A13-3225 | TX3c | 2013 | Somervell | TX | Raccoon | KF484526 |
| sm758 | 1T | 1984 | Bosque | TX | Skunk | KF484555 |
| sm741 | AR | 1984 | Nacogdoches | TX | Skunk | KF484549 |
| A11-3013 | TX1 | 2011 | Bastrop | TX | Skunk | KF484518 |
| sm744 | TX1 | 1984 | Galveston | TX | Skunk | KF484550 |
| sm749 | TX1 | 1984 | Fort Bend | TX | Skunk | KF484567 |
| sm750 | TX1 | 1984 | Austin | TX | Skunk | KF484568 |
| sm772 | TX1 | 1984 | DeWitt | TX | Skunk | KF484557 |
| TX4384 | TX1 | 1983 | Caldwell | TX | Skunk | KF484559 |
| sm761 | TX2as | 1984 | Hardeman | TX | Skunk | KF484569 |
| sm755 | TX2a | 1984 | Anderson | TX | Skunk | KF484553 |
| A11-6352 | TX2b | 2011 | Ellis | TX | Skunk | KF484561 |
| A12-9351 | TX2b | 2012 | Bowie | TX | Skunk | KF484519 |
| sm2528 | TX3b | 1994 | McCulloch | TX | Skunk | KF484543 |
| sm2590 | TX3b | 1994 | Tom Green | TX | Skunk | KF484544 |
| sm2600 | TX3b | 1999 | Tom Green | TX | Skunk | KF484545 |
| A12-9368 | TX3c | 2012 | Johnson | TX | Skunk | KF484562 |
| sm754 | TX3d | 1984 | San Saba | TX | Skunk | KF484552 |
| sm757 | TX3d | 1984 | Hills | TX | Skunk | KF484554 |
| sm759 | TX3d | 1984 | McCulloh | TX | Skunk | KF484556 |
| sm753 | TX3 | 1984 | Uvalde | TX | Skunk | KF484551 |
| A13-2198 | TX2b | 2013 | Rains | TX | Skunk | KF484524 |
| A13-2193 | TX2b | 2013 | Lamar | TX | Skunk | KF484522 |
| A13-2197 | TX1 | 2013 | Victoria | TX | Skunk | KF484565 |
| A13-2199 | TX3a | 2013 | Baylor | TX | Skunk | KF484566 |
| A13-2194 | TX3a | 2013 | Lubbock | TX | Skunk | KF484523 |
| A13-2196 | TX3a | 2013 | Young | TX | Skunk | KF484564 |
| A13-2195 | TX3c | 2013 | Lampasas | TX | Skunk | KF484563 |
| A13-4318 | TX3c | 2013 | Walker | TX | Skunk | KF484527 |
| A13-4320 | TX3c | 2013 | Brown | TX | Skunk | KF484528 |
| A13-4322 | TX1 | 2013 | Victoria | TX | Skunk | KF484530 |
| A13-4324 | TX3a | 2013 | Young | TX | Skunk | KF484532 |
| A13-4323 | TX3c | 2013 | Hill | TX | Skunk | KF484531 |
| A13-0712 | AZ | 2013 | Rio Rico | AZ | Skunk | KF484520 |
| CO8710 | Pre-GP | 1987 | Yuma | CO | Skunk | KF484536 |
| KS8640 | Pre-GP | 1984 | Hamilton | KS | Skunk | KF484540 |
| CO8948 | Pre-GP | 1987 | Yuma | CO | Skunk | KF484537 |
| AZ35930 | AZ | 1985 | Roosevelt | AZ | Skunk | KF484535 |
| AZ15763 | AZ | 1986 | Clifton | AZ | Skunk | KF484534 |
| MO1197 | MO | 1984 | Cole | MO | Skunk | KF484541 |
| A13-1418 | GP | 2013 | Pueblo | CO | Skunk | KF484521 |
| AR1030 | AR | 1979 | Clark | AR | Skunk | KF484533 |
| A13-4321 | TX3c | 2013 | Falls | TX | Skunk | KF484529 |
| G-0690 | TX2c | 1986 | Wise | TX | Unknown | KF484538 |
| ARSK090056 | MO | 2009 | 72560 | AR | Striped skunk | JX856083 |
| ARSK090057 | AR | 2009 | 71920 | AR | Striped skunk | JX856084 |
| ARSK090058 | AR | 2009 | 71923 | AR | Striped skunk | JX856085 |
| ARSK090059 | AR | 2009 | 71920 | AR | Striped skunk | JX856086 |
| ARSK090060 | AR | 2009 | 71920 | AR | Striped skunk | JX856087 |
| ARSK090062 | MO | 2009 | 72533 | AR | Striped skunk | JX856088 |
| ARSK090064 | AR | 2009 | 71920 | AR | Striped skunk | JX856089 |
| ARSK090065 | MO | 2009 | 72653 | AR | Striped skunk | JX856090 |
| ARSK090066 | MO | 2009 | 72774 | AR | Striped skunk | JX856091 |
| ARSK090067 | AR | 2009 | 75502 | AR | Striped skunk | JX856092 |
| ARSK090068 | AR | 2009 | 71920 | AR | Striped skunk | JX856093 |
| COSK090005 | GP | 2009 | 80836 | CO | Striped skunk | JX856044 |
| KSBC090011 | GP | 2009 | 67073 | KS | Bobcat | JX856050 |
| KSDG090015 | GP | 2009 | 66749 | KS | Domestic dog | JX856054 |
| KSRC090010 | GP | 2009 | 67443 | KS | Raccoon | JX856049 |
| KSRF090022 | GP | 2009 | 67635 | KS | Red fox | JX856061 |
| KSSK090001 | GP | 2009 | 67516 | KS | Striped skunk | JX856040 |
| KSSK090002 | GP | 2009 | 67560 | KS | Striped skunk | JX856041 |
| KSSK090003 | GP | 2009 | 67560 | KS | Striped skunk | JX856042 |
| KSSK090006 | GP | 2009 | 67520 | KS | Striped skunk | JX856045 |
| KSSK090012 | GP | 2009 | 67730 | KS | Striped skunk | JX856051 |
| KSSK090013 | GP | 2009 | 67226 | KS | Striped skunk | JX856052 |
| KSSK090014 | GP | 2009 | 67623 | KS | Striped skunk | JX856053 |
| KSSK090016 | GP | 2009 | 67584 | KS | Striped skunk | JX856055 |
| KSSK090017 | GP | 2009 | 67730 | KS | Striped skunk | JX856056 |
| KSSK090021 | GP | 2009 | 67749 | KS | Striped skunk | JX856060 |
| KSSK090027 | GP | 2009 | 66066 | KS | Striped skunk | JX856064 |
| KSSK090028 | GP | 2009 | 67530 | KS | Striped skunk | JX856065 |
| KSSK090030 | GP | 2009 | 66963 | KS | Striped skunk | JX856066 |
| KSSK090035 | GP | 2009 | 67749 | KS | Striped skunk | JX856068 |
| KSSK090050 | GP | 2009 | 67861 | KS | Striped skunk | JX856078 |
| KSSK090053 | GP | 2009 | 67572 | KS | Striped skunk | JX856080 |
| KSSK090054 | GP | 2009 | 67730 | KS | Striped skunk | JX856081 |
| KSSK090055 | GP | 2009 | 67226 | KS | Striped skunk | JX856082 |
| KSSK090084 | GP | 2009 | 67438 | KS | Striped skunk | JX856103 |
| KSSK090097 | GP | 2009 | 67749 | KS | Striped skunk | JX856110 |
| KSSK090100 | GP | 2009 | 67863 | KS | Striped skunk | JX856112 |
| MOSK090038 | MO | 2009 | 65655 | MO | Striped skunk | JX856070 |
| MOSK090041 | GP | 2009 | 64491 | MO | Striped skunk | JX856071 |
| MOSK090043 | MO | 2009 | 65775 | MO | Striped skunk | JX856073 |
| MOSK090098 | MO | 2009 | 65793 | MO | Striped skunk | JX856111 |
| NEEQ090004 | GP | 2009 | 68040 | NE | Horse | JX856043 |
| NEFE090018 | GP | 2009 | 68701 | NE | Domestic cat | JX856057 |
| NEFE090033 | GP | 2009 | 68601 | NE | Domestic cat | JX856067 |
| NERF090008 | GP | 2009 | 68416 | NE | Red fox | JX856047 |
| NESK090023 | GP | 2009 | 68601 | NE | Striped skunk | JX856062 |
| NESK090036 | GP | 2009 | 69032 | NE | Striped skunk | JX856069 |
| NESK090082 | GP | 2009 | 68745 | NE | Striped skunk | JX856102 |
| NESK090085 | GP | 2009 | 69101 | NE | Striped skunk | JX856104 |
| NESK090101 | GP | 2009 | 68822 | NE | Striped skunk | JX856113 |
| NESK090103 | GP | 2009 | 69101 | NE | Striped skunk | JX856115 |
| NESK090104 | GP | 2009 | 69045 | NE | Striped skunk | JX856116 |
| OKBV090073 | GP | 2009 | 73620 | OK | Cow | JX856095 |
| OKSK090075 | TX2b | 2009 | 73096 | OK | Striped skunk | JX856096 |
| OKSK090076 | TX2as | 2009 | 73521 | OK | Striped skunk | JX856097 |
| OKSK090078 | TX2b | 2009 | 73040 | OK | Striped skunk | JX856098 |
| OKSK090079 | TX2b | 2009 | 73456 | OK | Striped skunk | JX856099 |
| OKSK090080 | TX2b | 2009 | 73438 | OK | Striped skunk | JX856100 |
| MXSK3644 | MexSK-1 | 2009 | n/a | Mexico | Spotted Skunk | JQ685929 |
| MXSK13938 | MexSK-1 | 2007 | n/a | Mexico | Spotted Skunk | JQ685954 |
| TXML8 | MexSK-1 | 1986 | Brewster | TX | Mountain lion | KC792272 |
| A12_1992 | Rac | 2012 | n/a** | NC | Cat | KC792103 |
| A08_0591 | Rac | 2008 | n/a | NC | Beaver | KC791813 |
| A10_7349 | Rac | 2010 | n/a | PA | Cat | KC791862 |
| PA_R89 | Rac | 1989 | n/a | PA | Raccoon | U27217 |
| RRV ON-99-2 | Rac | 1999 | Ontario | Canada | Raccoon | EU311738 |
| WA1596 | EF-e1 | 2004 | n/a | WA | Bat | JQ595324 |
| TX2356 | LB | 2004 | n/a | TX | Bat | JQ595309 |
| FL148 | TB | 2004 | n/a | FL | Bat | JQ595321 |
| WA2020 | MY | 2005 | n/a | WA | Bat | JQ595328 |
| MXSK3636 | MXSK-2 (canine RABV) | 2009 | n/a | Mexico | Spotted Skunk | JQ685975 |

* “Skunk” indicates unidentified skunk species.

** n/a – not applicable
