# Supplementary figures and images for: The Phylogeography and Spatiotemporal Spread of South-Central Skunk Rabies Virus
Source: PLoS One. 2013 Dec 3;8(12):e82348. doi: 10.1371/journal.pone.0082348 (PMC3849458; doi:10.1371/journal.pone.0082348)

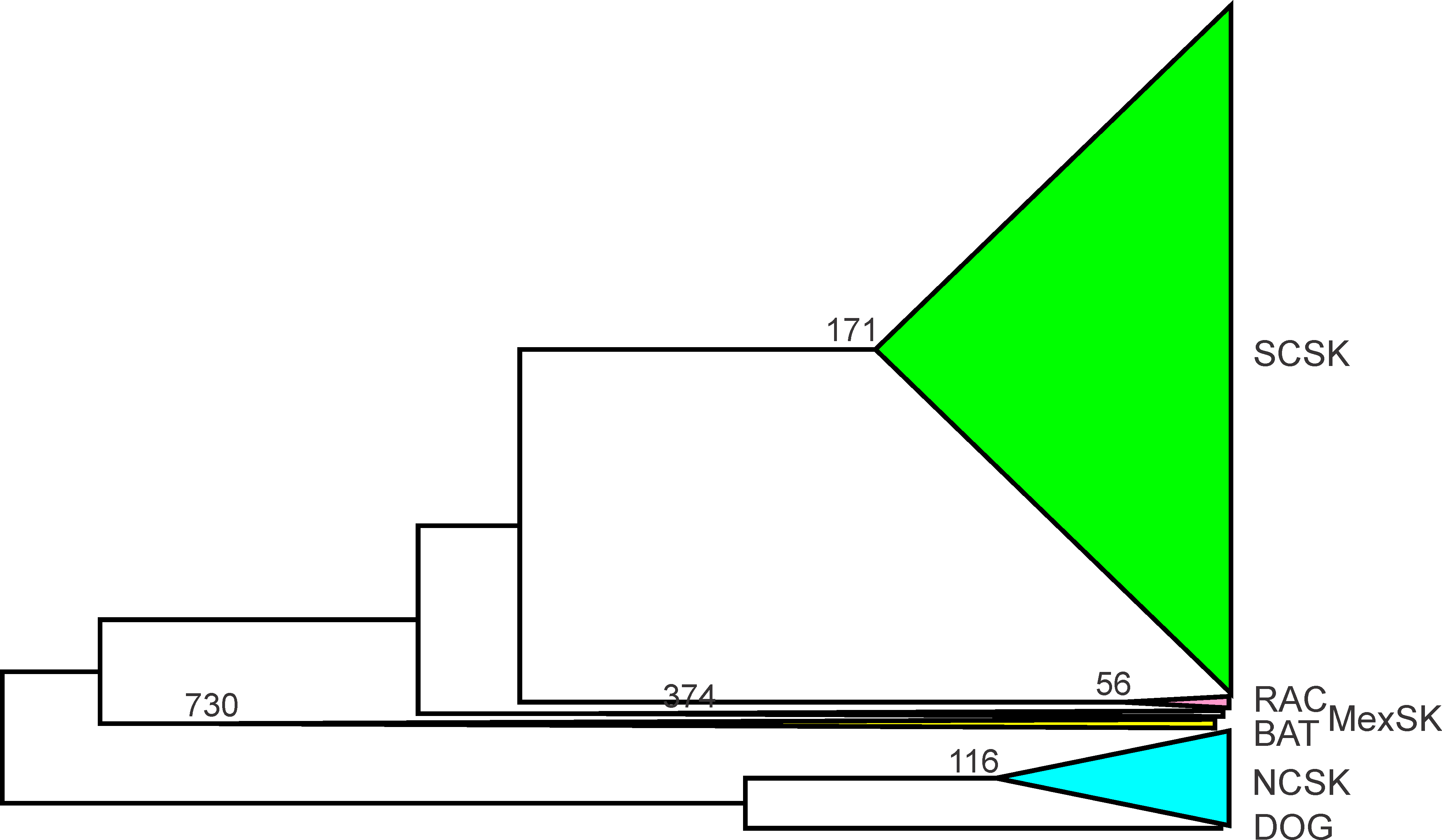

Supplement: Figure S1 — Preliminary estimates of the TMRCA for several North American RABV lineages based on complete and partial G gene sequences. The SCSK, raccoon (RAC), and Mexican skunk (MexSK) clusters include the same sequences that were used in this study (Figure 1). Bat viruses (BAT) include several representative viral sequences from various bat species. The north-central skunk (NCSK) cluster includes 35 dated viral sequences available from GenBank or generated de novo. (TIF) [file pone.0082348.s002.tif]
